# Unexpected Mutations in HIV-1 That Confer Resistance to the Tat Inhibitor Didehydro-Cortistatin A

**DOI:** 10.1128/mBio.01547-19

**Published:** 2019-07-09

**Authors:** Andrew P. Rice

**Affiliations:** a Department of Molecular Virology, Baylor College of Medicine, Houston, Texas, USA

**Keywords:** didehydro-cortistatin A, HIV-1, Tat, deep latency, latency

## Abstract

Didehydro-cortistatin A (dCA) is a human immunodeficiency virus type 1 (HIV-1) Tat inhibitor that functions by selectively binding to the RNA binding domain of Tat. In addition to inhibiting viral replication, dCA can drive HIV-1 into a state of “deep latency” in which latent viruses are refractory to reactivation. Mousseau et al. (G. Mousseau, R. Aneja, M. A. Clementz, S. Mediouni, et al., mBio 10:e01750-18, 2019, https://doi.org/10.1128/mBio.01750-18) have now selected dCA-resistant (dCA^r^) viruses *in vitro*. Remarkably, dCA^r^ viruses do not contain mutations in Tat or the viral transactivation-responsive element (TAR) RNA element that is targeted by Tat. Rather, the viruses contain a combination of mutations in the viral long terminal repeat (LTR) and Nef and Vpr proteins that result in an increase in basal RNA polymerase II (Pol II) transcription of integrated HIV-1. Interestingly, dCA^r^ viruses may be deficient in the establishment of latent infection because of their elevated basal Pol II transcription. dCA holds promise for strategies to achieve a functional cure of HIV-1 infection and justifies efforts to develop additional Tat inhibitors.

## Abstract (precis)

Didehydro-cortistatin A (dCA) is a human immunodeficiency virus type 1 (HIV-1) Tat inhibitor that functions by selectively binding to the RNA binding domain of Tat. In addition to inhibiting viral replication, dCA can drive HIV-1 into a state of “deep latency” in which latent viruses are refractory to reactivation. Mousseau et al. (G. Mousseau, R. Aneja, M. A. Clementz, S.

## TEXT

The human immunodeficiency virus type 1 (HIV-1) Tat protein was discovered more than 30 years ago as an activity in infected cells that potently enhanced gene expression directed by the viral long terminal repeat (LTR) sequences (1). Broad interest in Tat arose from subsequent discoveries that its *cis*-regulatory element is a viral RNA element and that it functions to activate RNA polymerase II (Pol II) transcriptional elongation rather than initiation—mechanisms of eukaryotic gene regulation that were unprecedented at the time of their discoveries (2). The finding that Tat is essential for HIV-1 replication and, therefore, might serve as an effective target for antiviral drugs further motivated interest in the viral protein (3). However, little progress was made in developing effective Tat inhibitors until 2012, when the Valente laboratory reported that didehydro-cortistatin A (dCA), an analog of the natural steroidal alkaloid cortistatin A, is a potent Tat inhibitor with an half-maximal effective concentration (EC_50_) in the low nanomolar range (4).

**FIG 1:**
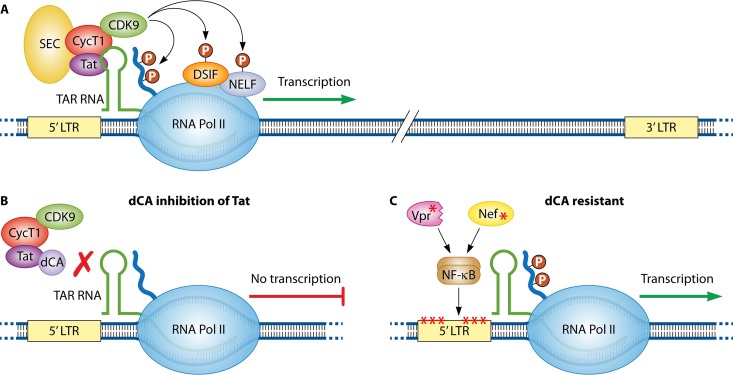
(A) Tat recruits cyclin T1/CDK9 to the TAR RNA element at the 5′ ends of viral transcripts, and the Super Elongation Complex (SEC) associates with this protein-RNA complex. CDK9 phosphorylates negative factors (DSIF and NELF) that restrict RNA polymerase II elongation. CDK9 also phosphorylates the carboxyl-terminal domain of RNA polymerase II and thereby creates binding sites for factors involved in RNA processing. (B) Didehydro-cortistatin A (dCA) binds to the RNA binding domain of Tat and prevents Tat/cyclin T1/CDK9 from binding to TAR RNA. (C) Viruses selected for resistance to dCA contain mutations in the 5′ LTR that increase the basal rate of RNA polymerase II transcription. dCA-resistant viruses also contain mutations in Nef and a mutation that truncates Vpr, and these mutant proteins enhance NF-κB activity.

The mechanism of action of Tat is now known in considerable detail (Fig. 1A). In the absence of Tat, Pol II initiates transcription from the 5′ viral LTR, but elongation is aborted due to the function of negative factors that restrict productive elongation. Tat contains two functional domains, an activation domain and an RNA binding domain. The activation domain binds to a general Pol II elongation factor termed P-TEFb that is composed of CDK9 and cyclin T1, while the RNA binding domain binds to the transactivation-responsive element (TAR) RNA stem-loop structure that forms at the 5′ ends of nascent viral transcripts. An additional protein complex, termed the Super Elongation Complex (SEC) (composed of ELL1/ELL2, AFF4, and ENL/AF9) associates with Tat/P-TEFb/TAR RNA (5). After assembly of this large protein complex, CDK9 phosphorylates multiple substrates in the Pol II complex, thereby overcoming the negative factors that restrict Pol II elongation. CDK9 also phosphorylates the carboxyl-terminal domain of the large subunit of Pol II, thereby providing binding sites for factors involved in RNA processing. Biochemical studies have shown that dCA binds selectively to a stretch of basic residues in the Tat RNA binding domain (6), and this is thought to prevent Tat from binding to TAR RNA and the assembly of the Tat/TAR RNA/P-TEFb/SEC complex (Fig. 1B).

The identification of dCA as an anti-Tat compound was fortuitous, as it was originally evaluated as a potential inhibitor of CDK11, a cellular factor involved in 3′ end processing of HIV-1 RNA (4). Given this background, there has been concern that the antiviral activity of dCA may not be due to its inhibition of Tat, but rather its effect on some cellular factor that manifests as an inhibition of Tat. However, the Valente laboratory has now identified and characterized HIV-1 isolates that were selected *in vitro* for resistance to dCA (7). The ability to select for viruses that contain mutations that confer resistance to dCA (dCA^r^ viruses) provides compelling evidence that dCA does indeed target a viral function, i.e., Tat, rather than some cellular function. The genetic modifications that confer resistance to dCA are surprising and have implications for strategies to achieve a functional cure of HIV-1 infection.

It was no easy task to select for viruses resistant to dCA. Attempts to select for dCA^r^ viruses were unsuccessful in CEM-SS and Jurkat CD4^+^ T cell lines, presumably reflecting a high genetic barrier to escape inhibition of a molecule that prevents Tat binding to TAR RNA. However, dCA^r^ virus populations were selected against the NL4-3 viral isolate in HeLa-CD4 cells over a 12-month period by passaging culture supernatants from an initial inoculum each week onto naive cells in the presence of increasing amounts of dCA. Two independent population of dCA^r^ viruses were obtained by this strategy. Although the viruses underwent selection in HeLa cells, the viral populations are resistant to dCA in CEM-SS and Jurkat cell lines, as well as primary CD4^+^ T cells.

The two populations of dCA^r^ viruses replicate to significantly higher levels than the parental virus in cell lines and primary CD4^+^ T cells. Chromatin immunoprecipitation (ChIP) experiments indicate that recruitment of Pol II to the viral LTR is enhanced in the dCA^r^ viruses, suggesting that the basal rate of Pol II transcription is elevated in dCA^r^ viruses. Surprisingly, deep sequencing revealed that the dCA^r^ viral populations contained no mutations in the Tat protein or the TAR RNA element. The absence of these mutations may reflect the fact that escape from dCA requires mutations in both the viral protein and RNA element, and the genetic barrier for this combination of mutations may be too high to overcome under the conditions used for selection. However, the dCA^r^ viral populations contain a number of mutations in the viral LTR, as well as mutations in the Gag, Pol, Vif, Vpr, Env, and Nef proteins.

To identify which mutations confer resistance to dCA, molecular viral clones were constructed from the dCA^r^ viral populations. The results from these clones indicate that acquisition of dCA resistance is multifactorial, involving mutations in the LTR, as well as mutations in Nef and a mutation that truncates Vpr after residue 57. The mutations in Gag, Pol, Vif, and Env in the dCA^r^ viral populations are apparently not involved in resistance to the Tat inhibitor.

Analysis of LTR reporter constructs further shows that the basal level of expression of dCA^r^ LTRs is significantly higher than the wild-type LTR. Mutations in the LTR include three point mutations upstream of the viral enhancer, three point mutations in the core promoter, and a duplication of an NF-κB/Sp1 binding site. The analysis of protein expression vectors indicates that both the mutant Nef and Vpr proteins enhance NF-κB activity, presumably contributing to elevated basal Pol II transcription by increasing NF-κB binding to its cognate sites in the viral LTR (Fig. 1C). Although the mechanisms whereby the mutant Nef and Vpr proteins enhance NF-κB remain to be determined, previous studies have reported that the viral proteins can affect NF-κB activity (8, 9). In summary, the data from dCA^r^ viral clones indicate that a combination of mutations in the LTR, Nef, and Vpr result in a significant increase in basal Pol II transcription initiation of integrated HIV-1, thereby removing the requirement for Tat to stimulate Pol II elongation. In other words, a large enough increase in transcriptional initiation conferred by the dCA^r^ mutations overcomes the block to transcriptional elongation that normally requires Tat function.

An intriguing property of dCA is its ability to drive HIV-1 into a state termed “deep latency.” When cell lines or patients’ cells harboring latent HIV-1 are treated with dCA for extended times, reactivation of virus is impaired upon discontinuation of dCA, and this impairment can be stable for a considerable time (10). ChIP assay results indicate that long-term dCA treatment leads to the establishment of a repressive chromatin state for integrated HIV-1 viruses, rendering them unresponsive to reactivation stimuli. These observations have led to the proposal that dCA might be used in a “block-and-lock” strategy; that is, in combination with current antiviral drugs, dCA may inhibit viral replication and “lock” the residual viral reservoir into a stable off-state, similar to the off-state of the majority of endogenous retroviral elements in the human genome. It is hoped that after an extended treatment time, dCA and other antivirals can be discontinued, and the viral reservoir will not reactivate to a level that cannot be controlled by the immune system, resulting in a functional cure of infection.

The phenotype of dCA^r^ viruses has implications for block-and-lock strategies. The dCA^r^ viruses have overcome the Tat inhibitor by ramping up basal Pol II transcription of the HIV-1 provirus and thereby increasing the overall production of viral proteins. The Mousseau et al. publication found that primary CD4^+^ T cells infected with dCA^r^ viruses are highly susceptible to killing by CD8^+^ T cells, as they present abundant viral antigen targets (7). Thus, dCA^r^ viruses may be deficient in the establishment of latent infection due to their increase in the basal level of expression of viral proteins that can be recognized by the immune system.

So what is next with dCA? The compound is expensive to synthesize and this has its limited availability. Hopefully, chemical synthesis of dCA can be optimized and scaled up, allowing the compound to be available to researchers in the field. The pharmacokinetic properties of dCA are favorable for an effective antiviral—indeed, it has been shown to be effective in inhibiting HIV-1 replication in humanized mice (11). An important next step will be to evaluate the efficacy of dCA in a simian immunodeficiency virus (SIV)/simian-human immunodeficiency virus (SHIV) rhesus macaque model. It will be crucial to determine whether dCA can inhibit SIV/SHIV replication in macaques and also establish deep latency in the animals. dCA inhibits SIV *in vitro*, presumably due to the conserved RNA binding domain in the SIV Tat protein (12). Therefore, there is optimism that dCA will be effective in rhesus macaques *in vivo*. It will be interesting to determine whether dCA^r^ SIV/SHIV viruses will arise in rhesus macaques *in vivo*, and if so, whether their genetic modifications will be similar to those selected for HIV-1 in human cells *in vitro*. dCA continues to hold potential as a component of a strategy to achieve a functional cure of HIV-1 infection. Furthermore, the findings with dCA provide a strong justification for the development of additional Tat inhibitors.
